# Predictors of adherence to an internet-based cognitive behavioral therapy program for individuals with chronic pain and comorbid psychological distress

**DOI:** 10.1186/s40359-021-00663-x

**Published:** 2021-10-12

**Authors:** Nils Gasslander, Sven Alfonsson, Amanda Jackalin, Cecilia Tengberg, Johanna Håkansson, Linda Huotari, Monica Buhrman

**Affiliations:** 1 Present Address: Department of Psychology, Uppsala University, P.O. Box 1225, 751 42 Uppsala, Sweden; 2 Centre for Psychiatry Research, Department of Clinical Neuroscience, Karolinska Institute & Stockholm Health Care Services, Stockholm County Council, Stockholm, Sweden

**Keywords:** Adherence, Internet, Cognitive behavioral therapy, Chronic pain, Depression, Disability

## Abstract

**Background:**

The burden caused by chronic pain is significant, affecting at least 10 percent of the world´s population. While internet-based treatments based on cognitive behavioral therapy (CBT) have been shown to be promising in this area, attrition levels vary significantly. The purpose of this study was to investigate predictor variables for participants’ adherence to an internet-based CBT treatment for individuals with chronic pain as well as to investigate associations between adherence and treatment outcome.

**Methods:**

Data for this study was retrieved from a randomized controlled trial including 95 individuals with chronic pain who received internet-based CBT. Treatment adherence was studied through three outcome variables: treatment progress, treatment completion and exercise completion. The predictor variables were grouped into four clusters: background variables (age, gender, marital status, level of education, and typical computer usage); the second cluster included health status variables (sick leave, current psychiatric diagnosis, previous psychotherapy for pain, current pharmacological treatment, previous depression, current depression, and current depressive symptoms); the third cluster included pain-related variables (opioid medication, history of pain, and pain symptoms) and the fourth cluster included motivation variables (measured with treatment preference, treatment credibility, compliance to the treatment schedule and contact with the therapists).

**Results:**

Findings showed that treatment progress was predicted by higher treatment credibility at baseline, whereas participants who were behind schedule in the second week of the program finished fewer treatment modules. When analyzing each cluster of predictor variables separately, current depressive symptoms also predicted fewer completed treatment modules**.** Among the pain-related variables, higher pain acceptance was the only predictor for completing more treatment modules. Treatment completion (which in this study was defined as having completed at least 75% of treatment modules) was predicted by higher treatment credibility and fewer depressive symptoms at baseline, and was thus similar to the results regarding treatment progress. Finally, all adherence variables predicted the treatment outcome pain interference.

**Conclusions:**

Low treatment credibility, depressive symptoms and falling behind the treatment schedule early on were the most important predictor variables for low treatment adherence, while a number of demographical and pain-related variables were not related to adherence. The results from this study may help clinicians identify patients who are less likely to complete, and thus benefit from, their pain treatment.

*Trial registration* ClinicalTrials.gov NTC03316846.

## Background

Chronic pain is a disabling condition that severely lowers the quality of life for many people and is very costly for society [1, 2]. While chronic pain can seldom be alleviated completely by medication or behavioral intervention, pain management programs can often ameliorate the pain symptoms and increase the quality of life for many patients. A range of programs based on cognitive behavioral therapy (CBT) have been shown to be effective in this regard. Individuals who are treated with CBT experience less pain and distress, though effect sizes are typically small to moderate [3]. The demand for these types of treatments is greater than what is currently available or possible to provide within strained health service budgets [1]. Internet-based CBT (iCBT) pain management programs may be more accessible and cost-effective. Previous studies have shown that iCBT can be effective in ameliorating pain-related symptoms such as catastrophizing, emotional distress and disability [4, 5]. However, not all patients benefit from these programs and some patients show low adherence, or discontinue their treatment for various reasons [4]. The precise relationship between treatment adherence and treatment outcomes in iCBT is still unclear, but it can be assumed that at least some level of adherence is necessary for the treatment to have an effect.

Treatment adherence in iCBT studies is often assessed by measuring the degree to which participants access the treatment, for example for how often the participants log on to a treatment webpage. Arguably, this is a rather crude measurement as it can be assumed that mere access to a treatment program is not enough and that participants need to engage with exercises or assignments in order to benefit from the treatment. In a previous study of participants in an online stress management program, it was found that completion of treatment exercises was more strongly associated with positive treatment effects than more generally accessing the program [6]. This is largely congruent with face-to-face CBT, for which it has been shown that completing exercises or assignments is associated with positive treatment outcomes [7]. In CBT it is assumed that translating what is learnt in therapy into daily behavioral changes is essential, and overall that behavior change is important for positive outcomes [8].

The focus in CBT is to train patients to use cognitive and behavioral strategies designed to reduce psychological distress and minimize the negative impact of pain on daily function. Maladaptive cognitions and behaviors that contribute to the maintenance of disability are targeted. Different cognitive and behavioral techniques are presented; such as cognitive restructuring, applied relaxation, exposure to pain symptoms (controlled exposure to painful sensations in order to facilitate behavioral flexibility) and education about the effects of chronic pain in the person’s daily life [9]. In the last decade, there has been growing interest in the effectiveness of Acceptance and Commitment Therapy (ACT) and other third-generation CBT interventions. An important aspect of ACT is the awareness and non-judgmental acceptance of all experiences, both negative and positive, as well as identification of valued life directions and committed action toward goals that support those values. ACT focuses on improving functioning and decreasing interference of pain with value-driven action [10, 11].

The treatment in the present study was based on the CBT and ACT-techniques described above and targeted various forms of psychological distress such as depressive symptoms, anxiety and insomnia. The treatment was delivered through the internet and participants received instruction in different CBT techniques tailored to the participants reported symptoms; e.g., behavioral activation, techniques to manage worry, applied relaxation, and exposure exercises. A more detailed description of the treatment content will be published separately [12].

Previous studies on iCBT have shown that both treatment adherence and treatment outcomes vary considerably [4], and it has been shown that variables such as gender as well as psychiatric symptoms may predict who will adhere to as well as benefit from iCBT [13]. Investigating predictor variables for treatment adherence may help in early identification of patients who may need additional support or who are less likely to benefit from the treatment format. If iCBT is to be offered to a wider range of patients, there is a need to not only investigate whether the programs are effective but also for whom they are effective and whether some patients should be offered alternative formats.

The aim of the present study was to investigate predictor variables for adherence to an internet-based CBT program for individuals with chronic pain and psychological distress, as well as to investigate associations between adherence and treatment outcomes. Since, to our knowledge, no previous study has investigated predictors for adherence in this patient group, this study included a large number of variables related to participants backgrounds, health, pain and motivation.

## Methods

### Design, procedure and participants

Data for this study was retrieved from a previously conducted iCBT study for individuals with chronic pain [12]. Participants were recruited from a specialist pain clinic at the Uppsala University Hospital in Sweden. Inclusion criteria required that participants had experienced pain for more than three months, reported at least one form of psychological distress, had been medically assessed for their pain condition within one year before the screening was conducted, had access to a personal computer with internet access, and had the ability to read and write Swedish fluently (as the treatment material was written in Swedish). Patients were excluded if they were currently undergoing or planning to start CBT during the course of the study; if they had made major changes to their medications during the last two months; had planned surgical interventions during the timeframe of the study, or reported significant symptoms of severe depression (> 16 points in the M.I.N.I. suicide assessment section with clinical assessment); psychosis (met M.I.N.I. criteria for ongoing psychotic episode), or substance abuse (met M.I.N.I. criteria for ongoing substance abuse disorder). In addition, participants who did not log in to the treatment platform to fill out the baseline measurements were excluded.

After a structured screening and initial technical assistance via telephone, a total of 187 participants fulfilled the inclusion criteria and were randomized to either iCBT (n = 95) or a waiting list control group (n = 92). The present study only included data from the iCBT group (n = 95). The intervention consisted of 6–13 modules, each designed to be completed in one week. Treatment content was individualized by assigning modules to each participant targeting the specific psychological issues described by the participant in the initial screening. All modules included written information and instructions related to the current module’s theme and concluded in 1–3 practical exercises. Content was in the form of text, images and audio. Participants were asked to report the results of their exercises at the end of each module. Their assigned therapist then reviewed and provided feedback (mainly consisting of positive reinforcement and answers to questions) within 24h on weekdays, using a secure messaging function on the treatment platform. Therapist assignment was randomized, and therapists were four students in the fifth year of a five-year clinical psychologist professional degree program, as well as one licensed psychologist. Therapists were supervised by a licensed psychologist and psychotherapist with extensive experience in treating chronic pain. All therapists had CBT training and clinical experience. The treatment content and all measurements were delivered online though the treatment platform developed at the Uppsala University Hospital. Participants were asked to fill out a battery of self-report questionnaires before, during, and after the intervention; All 95 participants in the iCBT-group provided data from pre-treatment assessments. Follow-up data was gathered for the intervention group 12 months after the end of treatment.

### Outcome variables

Treatment adherence was divided into three variables labeled treatment progress, treatment completion and exercise completion. These three variables aimed to investigate both the more superficial aspect of adherence, corresponding to working one’s way through the treatment modules and finishing the treatment program, as well as a deeper aspect of adherence corresponding to actively working with the exercises included in the treatment program to a higher extent. Consequently, treatment progress was assessed by counting the proportion of the assigned number of treatment modules each participant had completed at the end of the treatment period (range 0–100%). Treatment completion was assessed by assessing each participant’s progress through the program and coding 0 if the participant had completed < 75% of the modules and 1 if the participant had completed ≥ 75% of the modules at the end of the study. Exercise completion was assessed by counting the proportion of the assigned number of completed exercises for each participant over the whole treatment (range 0–100%). Exercises were smaller optional tasks meant to facilitate engagement and comprehension of the material, and were designed to take no more than 5 min to complete. Treatment outcome was measured with the MADRS-S and the MPI Pain interference scales (please see below) that were used as the primary outcomes in the original study [12]. For both outcome variables, the change score between pre- and post-treatment was calculated.

### Predictor variables

A review of published research showed that there are few consistent predictor variables for adherence and outcomes of pain treatment [14]. Variables that have been found to predict outcomes in some (but not all) studies include: gender, age, level of education, baseline pain severity and disability, coping style, and psychiatric symptoms [14, 15]. Regarding internet-based treatment, a few variables have consistently been associated with treatment outcomes, including psychiatric symptoms and treatment credibility, while results regarding demographical variables are mixed [16–19]. Based on the research literature and our clinical experience working with this patient population, potential predictor variables were identified and grouped into four clusters. The first cluster included the background variables; age (years), gender (female/male), marital status (married or cohabitant/single), level of education (high school/university), and estimated computer usage (hours per day). The second cluster included the health status variables; sick leave (number of days last year), current psychiatric diagnosis (yes/no), previous psychotherapy for pain (yes/no), current depression (yes/no), and current depressive symptoms (MADRS-S, Montgomery-Åsberg Depression Rating Scale) [20]. The third cluster included the pain-related variables opioid medication (yes/no), history of pain (years), pain acceptance (CPAQ, Chronic Pain Acceptance Questionnaire) [21] and pain symptoms (CPAQ, MPI, Multidimensional Pain Inventory) [22]. The fourth cluster included the motivation variables; treatment preference (the participant’s preferred treatment content, see below), treatment credibility (TCS, Treatment Credibility Scale) [23, 24], and whether the participant followed the treatment schedule the first two weeks (yes/no). The characteristics of each predictor variable are described in “Appendix”.

Background and health status variables were collected at the structured screening interview by phone, with the exception of current depressive symptoms which was measured with the MADRS-S [20], filled out digitally by the participants. The MADRS-S is a 9-item self-report version of a questionnaire measuring depressive symptoms on a scale from 0 to 6, where higher values indicate a higher degree of depressive symptoms.

Pain-related variables (opioid medication and history of pain) were also collected during the screening interview via phone. However, the pain-related instruments CPAQ [21] and MPI [22], were answered digitally. The CPAQ [21] is a questionnaire assessing pain acceptance on 20-items divided in two subscales: pain willingness (willingness to experience pain, rated from 0 to 54) and activity engagement (engaging in activities despite the pain, rated from 0 to 66). The MPI [22] measures psychosocial and behavioral consequences of chronic pain. The present study used the Swedish version of the first section of the MPI [25], consisting of 22 items and five subscales with scores ranging from 0 to 6: pain severity (current degree of pain), pain interference (how much pain interferes with daily functioning), life control (perceived control over ones’ life), affective distress (level of emotional distress) and social support (perceived support received by others).

Treatment motivation variables were collected using information from the screening phone interview; by recording treatment logs, messages and timestamps; as well as the instrument TCS [23, 24]. The variable treatment preference consisted of 11 categories (pain coping, pain relief, acceptance, anxiety, depression, relationships, sleep, tension, stress, knowledge, and other) aimed to capture participants’ needs by asking the participants about their distress and hopes for the treatment in the initial interview. The TCS measures perceived credibility of a treatment with 5-items asking questions such as “How logical does this treatment seem to you?”, with the participants answering each question on a scale of 1 (“Not at all”) to 10 (“Very”).

### Analysis

Before analysis, data was screened for outliers, and normality, linearity, and homoscedasticity was evaluated by examining residual scatterplots between predicted variables and errors of prediction. These were found to deviate for a number of variables: sick-leave showed a bimodal distribution and was therefore split at the median and recoded into a dichotomous variable, and the variables computer time and history of pain were found to be skewed and were therefore log-transformed. Because subscales were entered into the analyses, multicollinearity was assessed by analyzing the variance inflation factor for each predictor variable, and found to be non-problematic.

Bivariate regression analysis was first used to identify candidate (*P* < 0.10) predictor variables for each adherence variable. All identified predictor variables were included in subsequent multiple regression analyses using a backward deletion process for each adherence variable. Then, bivariate regression analysis was used to investigate whether the adherence variables could significantly predict the two treatment outcome variables, while controlling for pre-treatment scores. Linear regression was used for the continuous outcome variables treatment progress and exercise completion, and logistic regression was used for the dichotomous outcome variable treatment completion. Linear regression was used for the two treatment outcome variables. Cox-Snell *R*^*2*^ and Nagelkerke *R*^*2*^ were used as a measure of overall model fit in the logistic regression analyses, and *R*^*2*^ was used in the linear regression analyses. Because some of the variables had distributions that deviated somewhat from normality, the final regression models were confirmed using robust regression analyses with bootstrap and bias correction. The sample size of 95 was deemed adequate for regression analysis of a maximum of seven predictor variables for each outcome variable. A *p* value of 0.05 was considered the threshold for statistical significance if not stated otherwise, whereas exact *p* values were reported for the final analyses.

## Results

### Treatment progress

In the first bivariate phase of analysis, the following predictor variables for number of completed treatment modules were identified; sick-leave, depressive symptoms (MADRS-S), pain acceptance (CPAQ Willingness), MPI Life control, late second week, and treatment credibility (TCS). In the subsequent multiple regression analysis, late second week (B = − 0.20, SE = 0.08, 95% CI = − 0.35 to − 0.05, β = − 0.33, t = 2.72, p = 0.009), depressive symptoms (B = − 0.01, SE = 0.01, 95% CI = − 0.02 to − 0.01, β = − 0.41, t = 3.43, p = 0.001) and treatment credibility (TCS, B = 0.01, SE = 0.01, 95% CI = 0.01–0.02, β = 0.25, t = 2.11, p = 0.040) remained as significant predictor variables (*R*^2^ = 0.28). Analyzing each cluster of predictor variables separately showed that depressive symptoms (MADRS-S) was a significant health-related predictor while pain acceptance (CPAQ Willingness) was a significant pain-related predictor, and late second week and treatment credibility (TCS) were significant engagement predictors for number of completed treatment modules. Please see Table 1 for predictor cluster details.

**Table 1:** Predictor variables within each cluster for number of completed treatment modules (n = 95)

Completed treatment modules	B (SE)	95% CI	β	t	P
Health variables					
Sick-leave	− 0.09 (.09)	− 0.27; 0.09	− .12	0.97	.337
MADRS-S	− 0.01 (0.01)	− 0.02; − 0.01	− .36	2.93	.005
Pain variables					
MPI Life Control	0.05 (0.04)	− 0.02; 0.12	.15	1.38	.170
CPAQ Willingness	0.01 (0.01)	0.01; 0.02	.32	3.04	.003
Engagement variables					
Late second week	− 0.16 (0.07)	− 0.30; − 0.02	− .26	2.22	.030
TCS	0.01 (0.01)	0.00; 0.02	.24	2.07	.043

*MADRS* Montgomery-Åsberg Depression Rating Scale, *MPI* Multidimensional Pain Inventory, *CPAQ* Chronic Pain Acceptance Questionnaire, *TCS* Treatment Credibility Scale

### Treatment completion

In the bivariate phase of the logistic regression analysis, sick-leave, pain acceptance (CPAQ Willingness), depressive symptoms (MADRS-S), pain interference (MPI Interference subscale), and treatment credibility (TCS) were significant predictors for completing the treatment. Comparing the models from multivariate logistic regression analyses comprising these variables resulted in a final model including only MADRS-S and TCS (*R*^2^ = 0.17/0.23), see Table 2.

**Table 2:** Predictor variables for treatment completion (n = 95)

Completed treatment	B (SE)	W	p	OR	95% OR
MADRS-S	− .08 (.03)	8.03	.005	.92	.86; .97
TCS	.08 (.03)	5.16	.023	1.08	1.01; 1.15

*MADRS* Montgomery-Åsberg Depression Rating Scale, *TCS* Treatment Credibility Scale

### Completed exercises

In the bivariate phase of analysis, late second week and treatment credibility were identified as predictor variables for number of completed treatment exercises. In the subsequent multiple regression analysis only treatment credibility remained as a significant predictor for completed exercises (*R*^2^ = 0.15), see Table 3.

**Table 3:** Predictor variables for number of completed exercises (n = 95)

Completed treatment exercises	B (SE)	95% CI	β	t	P
Late second week	− 0.08 (0.05)	− 0.18; − 0.02	− .19	1.69	.096
TCS	.01 (.001)	0.01; .0.02	.34	2.99	.004

*TCS* Treatment Credibility Scale

### Treatment outcome

Change in depressive symptoms could not be significantly predicted by the adherence variables treatment progress (B = 4.39, SE = 2.43, 95% CI = − 0.46 to 9.24, β = 0.23, t = 1.81, p = 0.076), treatment completion (B = 1.64, SE = 1.66, 95% CI = − 1.67 to 4.95 β = 0.13, t = 0.99, p = 0.33), or completed exercises B = 3.84, SE = 3.96, 95% CI = − 0.2.75 to 10.43, β = 0.15, t = 1.17, p = 0.25).

Change in pain interference could be significantly predicted by treatment progress (B = 0.78, SE = 0.32, 95% CI = 0.15–1.41, β = 0.30, t = 2.47, p = 0.016), treatment completion (B = 0.59, SE = 0.21, 95% CI = 0.16–1.02, β = 0.33, t = 2.76, p = 0.008) and completed exercises (B = 0.84, SE = 0.03, 95% CI = 0.02—0.14, β = 0.33, t = 2.81, p = 0.007).

In a post-hoc bivariate regression analysis, treatment credibility was a significant predictor for both change in depressive symptoms (B = 0.27, SE = 0.10, 95% CI = 0.06–0.47, β = 0.30, t = 2.76, p = 0.012) and pain interference (B = 0.03, SE = 0.14, 95% CI = 0.01–0.06, β = 0.28, t = 2.32, p = 0.005).

## Discussion

The aims of this study were to investigate whether adherence to an internet-based CBT program could be predicted at baseline or early in treatment, and whether adherence in turn could predict treatment outcomes. Though measurements of adherence vary across studies, low adherence to a treatment suggests that a lower “dose” of received treatment content has been received, and could imply lower engagement and motivation. It is not surprising then, that greater adherence relates to improved treatment outcomes [16, 26–28], highlighting the importance of improving adherence to treatment. These results were partly replicated in the current study in which greater adherence was associated with improvement in pain interference but not in depressive symptoms. The reasons for these differences have not been clearly established. One possibility is that depressive symptoms are more affected by e.g., early treatment content, being in contact with a therapist, or other factors that were not assessed in the current study. Further, reducing pain interference requires more complex interventions that involve several aspects of an individual’s life. Accordingly, while pain interference was targeted in all modules of the present trial, depression was targeted in specific, early modules. The rationale for placing behavioral activation modules early in the treatment was that improvements in mood and a higher activity level were thought to have the potential to improve adherence to later treatment content. Additionally, several participants reported conflicting schedules or a lack of time as reasons of attrition, implying activities that might themselves have functioned as type of behavioral activation. This could be an explanation why interference was predicted by adherence and not depression.

In the present study, adherence was divided into three variables. The first, treatment progress, could be predicted by reported treatment credibility (TCS score) at baseline, depressive symptoms, and by the participant being behind schedule in the second week of the program. Treatment credibility has shown to be a reliable predictor for adherence to internet-based treatments in a number of studies [13] and will be further discussed below. As seen in previous studies, elevated depressive symptoms are a common risk factor for lower adherence to internet-based interventions. Our observation that participants who were behind schedule in the second week of the program also finished fewer treatment modules is not surprising since some of these participants continued to lag behind the recommended schedule and had difficulties in finishing the treatment during the study period, or decided to discontinue the treatment. Still, it is important to note that early signs of non-adherence to a treatment program seems to indicate that the participant will not follow through with the program. This pattern has been seen in other studies as well [29] and may help researchers or clinicians who work with these programs identify participants who are at risk of dropping out of the program. For example, participants who are not following the treatment program can be contacted for encouragement, troubleshooting, or for discussion of alternative treatment. When analyzing each cluster of predictor variables separately, depressive symptoms (MADRS-S score) were also a significant predictor for fewer completed treatment modules. Depressive symptoms include reduced initiative, motivation and executive function, which may make it more difficult to follow an internet-based treatment program that could be hypothesized to put higher demands on these faculties than standard face-to-face treatments. Based on these results, participants with elevated depressive symptoms may be better helped by other treatment alternatives. Among the pain-related variables, higher pain acceptance (CPAQ Willingness subscale) was the only predictor for completing more treatment modules. This may seem surprising since a low acceptance could be thought to motivate individuals to work with a treatment program for pain management. However, pain management programs, as the one used in this study [4, 5] are not centered on reducing the pain intensity as such, but on learning to live with pain. Thus, having a higher pain acceptance before the start of treatment may help in accepting this treatment premise.

Treatment completion, which in this study was equal to having completed at least 75% of the treatment modules, was predicted by depressive symptoms and treatment credibility, and was thus similar to the results regarding treatment progress. This is unsurprising given that the two variables overlap, but it is still important to note that treatment credibility and depressive symptoms are associated with both treatment progress and treatment completion. A similar pattern was seen for the third outcome variable, proportion of completed treatment exercises, though this variable was only predicted by treatment credibility.

As mentioned above, treatment credibility has been shown to be a predictor for adherence to internet-based treatments in previous studies, and these results were replicated in the present study [16]. Treatment credibility, as assessed with the TCS, seems to capture an important motivational factor that is consistently associated with treatment engagement. Unfortunately, this measure has only been used in internet-based studies, and it is unknown whether the same association can be seen in face-to-face treatments. Assessing treatment credibility was initiated early in the iCBT field, since it was hypothesized that credibility of this treatment format would be important for treatment outcomes [30]. However, treatment credibility is a broader variable and likely captures a participants’ overall belief that a treatment may be effective, thus promoting engagement. It seems plausible that a participant who believes that a treatment will be effective invests more time and energy in it than a participant who is more skeptical. Previous research has shown that treatment expectations is a consistent predictor variable for outcomes in psychotherapy in general. While it has been suggested that the phenomenon is closely related to the working alliance (another variable consistently associated with outcomes), the specific psychological processes remain unknown [31–33]. In this study, treatment credibility was also a significant predictor for treatment outcome, though it could not be assessed whether this association was mediated through treatment adherence.

A large number of predictor variables investigated in this study were found not to be significantly associated with treatment adherence. Background variables such as age, gender and education have in some previous studies been shown to be associated with treatment adherence and/or treatment outcomes, but these results were not replicated in this study [13]. It is possible either that the participants in the current study came from another clinical population compared to previous studies since background variables for patients with chronic pain may differ from other patient groups, or that the range of these variables was too restricted. A number of health- and pain-related variables were investigated as possible predictor variables in this study, and all but depressive symptoms and pain acceptance were discarded in the first bivariate analyses. This was somewhat surprising since we expected variables associated with overall health, pain burden and pain medication to be associated with daily function and treatment motivation. In contrast, these null-findings suggest that iCBT pain management may be offered to patients regardless of most background-, health- or pain-related variables.

While the effectiveness of iCBT programs has been shown in numerous studies, adherence to internet-based CBT programs varies. Unfortunately, many studies do not report treatment adherence and the associations between adherence and treatment outcomes are not well understood. For example, previous studies have suggested that completing the treatment assignments is more important than passively consuming the treatment program [6]. Further studies that investigate the mechanisms of iCBT programs is warranted, such as identification and investigation of essential components and features of such programs.

## Conclusions

This study investigated several variables that could be used as early predictors of adherence to an internet-based treatment for patients with chronic pain and psychological distress. Of these variables, treatment credibility, depressive symptoms and low engagement early on in the treatment were the most important factors. These factors could be of clinical use when assessing a patient’s likelihood of remaining in internet-based treatment, thus enabling clinicians to offer increased support or provide other treatment options early on. Further, several variables turned out to not predict treatment adherence, implying the possibility that internet-based treatment could be offered this patient group regardless of demographic factors or overall health. Finally, based on the results of the current as well as previous studies, it seems that internet-based treatments are most beneficial to those who perceive the programs to be effective and credible.

## Data Availability

The datasets analyzed during the current study are available from the corresponding author on reasonable request.

## Appendix

### Outcome variables

**Table:**

Variable	Description	n [%]	M [SD]	Range
Treatment progress (n = 95)	The proportion of modules completed* by each participant at the end of the treatment period		51.9 [37.3]	0–100
Treatment completion (n = 95)	Coded 0 if the participant had completed* < 75% of the modules and 1 if the participant had completed ≥ 75% of the modules at study end. Module completion was defined as having sent in all homework assignments for the relevant module	0 = 62 [65.3] 1 = 33 [34.7]		0–1
Exercise completion (n = 84)	The proportion of assigned exercises completed by each participant. “Exercises” were smaller optional tasks embedded in the theoretical parts of the treatment content, meant to facilitate engagement and comprehension of the material, as were designed to take 1–5 min to complete. E.g. reflecting on previous experience, or testing a short mindfulness task		75.1 (26.0)	0–100

*Completion was defined as having turned in all homework exercises.

### Predictor variables

**Table:**

Variable	Description	n [%]	M [SD]	Range
Age (n = 95)	Age (years) at time of interview		45.6 (11.1)	20–64
Gender (n = 95)	0 = Female	0 = 70 [73.7]		0–1
	1 = Male	1 = 25 [26.3]		
Marital status (n = 95)	0 = Single/divorced	0 = 28 [29.5]		0–1
	1 = Married/cohabitant	1 = 67 [70.5]		
Level of education (n = 95)	0 = Completed 9-year compulsory education	0 = 7 [7.4]		0–3
	1 = Completed upper secondary education	1 = 35 [36.8]		
	2 = Completed ≥ 2 years university education	2 = 16 [16.8]		
	3 = Completed > 2 years university education	3 = 37 [38.9]		
Computer usage (n = 94)	Typical hours spent per day		3.1 [2.8]	0–12
Sick leave (n = 76)	Number of days on sick leave last year		154.1 [161.4]	0–365
Current psychiatric diagnosis (n = 94)	0 = No ongoing psychiatric diagnosis according to MINI-interview (DSM-5 criteria)	0 = 41 [43.6]		0–1
	1 = One or more ongoing psychiatric diagnoses according to MINI-interview (DSM-5 criteria)	1 = 53 [56.4]		
Previous psychotherapy (n = 95)	0 = Have not previously received psychotherapy	0 = 18 [18.9]		0–1
	1 = Have previously received psychotherapy	1 = 77 [81.0]		
Current depression (n = 95)	0 = No ongoing Major depressive disorder according to MINI-interview (DSM-5 criteria)	0 = 37 [38.9]		0–1
	1 = Ongoing Major depressive disorder according to MINI-interview (DSM-5 criteria)	1 = 58 [61.0]		
Current depressive symptoms (n = 95)	Montgomery-Åsberg Depression Rating Scale (MADRS-S) score before treatment		20.5 [9.5]	2–45
Opioid medication (n = 94)	0 = not currently on opioid medication	0 = 43 [45.8]		0–1
	1 = currently using opioid medication	1 = 51 [54.3]		
History of pain (n = 95)	Years with chronic pain at time of interview		15.4 [11.1]	0.17–51
Pain acceptance (n = 95)	Chronic Pain Acceptance Questionnaire (CPAQ)		51.5 [19.4]	6–96
	AE = Activity Engagement		AE = 27.9 [13.1]	AE = 0–57
	PW = Pain Willingness		PW = 23.6 [8.5]	PW = 2–48
Pain symptoms (n = 95)	Multidimensional Pain Inventory (MPI)			Sev = 2–6
	Sev = Pain Severity		Sev = 4.0 [0.8]	
	Int = Pain Interference		Int = 4.5 [1.1]	Int = 1.8–6.1
	Lif = Life Control		Lif = 2.4 [1.1]	Lif = 0–4.8
	Aff = Affective Distress		Aff = 3.1 [0.7]	Aff = 1.3–4.7
	Sup = Social Support		Sup = 3.6 [1.7]	Sup = 0–6
Treatment preference (n = 88)	Participants preferred treatment content as reported in interview. Preferences was organized into 11 categories depending on the issues participants wanted help with			
1 = Coping with pain	1 = 32 [36.4]		1–11	
2 = Increased acceptance	2 = 8 [9.1]			
3 = Pain relief	3 = 7 [8]			
4 = Reduced worry, fear, or anxiety	4 = 27 [30.7]			
5 = Reduced depression, sadness, inactivity	5 = 32 [36.4]			
6 = Improved communication, or relationships	6 = 12 [13.6]			
7 = Improved sleep	7 = 16 [18.2]			
8 = Increased relaxation, calm	8 = 3 [3.4]			
9 = Reduced stress	9 = 16 [18.2]			
10 = Knowledge or information regarding pain	10 = 5 [5.7]			
11 = Other issues	11 = 5 [5.7]			
Treatment credibility (n = 94)	Treatment Credibility Scale (TCS) score before treatment		34.5 (8.0)	12–50
Following the treatment schedule the first two weeks (n = 69)	0 = Did not complete the second module of the iCBT-treatment within 2 weeks	0 = 27 [0.39]		0–1
	1 = Completed the second module of the iCBT-treatment within 2 weeks	1 = 42 [0.44]		

## Abbreviations

ACT: Acceptance and commitment therapy
CBT: Cognitive behavioral therapy
CPAQ: Chronic Pain Acceptance Questionnaire
iCBT: Internet-based cognitive behavioral therapy
MADRS: Montgomery-Åsberg Depression Rating Scale
MPI: Multidimensional Pain Inventory
TCS: Treatment Credibility Scale
